# The beneficial effects of a gas-permeable flask for expansion of Tumor-Infiltrating lymphocytes as reflected in their mitochondrial function and respiration capacity

**DOI:** 10.1080/2162402X.2015.1057386

**Published:** 2015-06-05

**Authors:** Marie-Andrée Forget, Cara Haymaker, Jennifer B Dennison, Christopher Toth, Sourindra Maiti, Orenthial J Fulbright, Laurence J N Cooper, Patrick Hwu, Laszlo G Radvanyi, Chantale Bernatchez

**Affiliations:** 1 Department of Melanoma Medical Oncology; The University of Texas MD Anderson Cancer Center (MDACC); Houston, TX USA; 2 Department of Systems Biology; The University of Texas MDACC; Houston, TX USA; 3 Division of Pediatrics; The University of Texas MDACC; Houston, TX USA; 4 Lion Biotechnologies; Tampa, FL USA; 5 Department of Immunology; H. Lee Moffitt Cancer Center; Tampa, FL USA

**Keywords:** TIL ACT, gas-permeable membrane, mitochondrial function, oxidative phenotype, tumor-infiltrating lymphocytes

## Abstract

Adoptive transfer of autologous *ex vivo* expanded tumor-infiltrating lymphocytes (TIL) is a highly successful cell therapy approach in the treatment of late-stage melanoma. Notwithstanding the success of this therapy, only very few centers worldwide can provide it. To make this therapy broadly available, one of the major obstacles to overcome is the complexity of culturing the TIL. Recently, major efforts have been deployed to resolve this issue. The use of the Gas-permeable flask (G-Rex) during the REP has been one application that has facilitated this process. Here we show that the use of this new device is able to rescue poor TIL growth and maintain clonal diversity while supporting an improved mitochondrial function.

## Abbreviations

ACT: Adoptive Cell Transfer
BTLA: B and T Lymphocyte Attenuator
ECAR: extracellular acidification rate
OCR: oxygen consumption rate
OXPHOS: oxidative phosphorylation activity
PBMC: peripheral blood mononuclear cells
REP: Rapid Expansion Protocol
TIL: Tumor-Infiltrating Lymphocyte.

## Introduction

Tremendous progress in the use of immunological approaches for cancer treatment was made in the last decade. Metastatic melanoma has served as a “poster child” in the development both cellular and humoral immunotherapy. Adoptive transfer of autologous *ex vivo* expanded TIL is a highly successful cell therapy approach in the treatment of late-stage melanoma with reported clinical response rates averaging around 50% in multiple phase II studies.^1-3^ Notwithstanding the success rate of adoptive transfer of TIL, the therapy is still only offered in clinical trials which are conducted in very few centers worldwide. To make this therapy broadly available, one of the major obstacles to overcome is the complexity of culturing the TIL.

As previously described, the expansion of TIL for ACT is traditionally viewed as a two-step process: the pre-rapid expansion protocol (REP) step and the REP step.^4^ The pre-REP step consists of the first outgrowth of TIL from tumor fragments seeded in 24 well plates in media containing IL-2. The REP is a 14 d process in which the cells are expanded with anti-CD3 antibody and IL-2 in T-175 flasks for the first 7 d and transferred into gas permeable bags for the remaining 7 d. Recently, Jin et al. published a study in which they replaced the entire REP setting by one unique device, a G-Rex.^5^ This G-Rex (Wilson-Wolf, Minneapolis, MN) showed a unique placement for oxygen uptake given the location of the membrane at the bottom of the flask where the cells are actually seeded.^6^ They strikingly demonstrated the technical advantages of these flasks in terms of high fold expansion of the TIL and ease of use which reinforced other centers like ours to move forward with testing these culture devices for our clinical TIL REP.^5^

During the establishment of our different culture parameters for the use of the G-Rex in a clinical setting, we noticed that the G-Rex could help to rescue poor lymphocyte growth when compared to TIL growth using the traditional culture devices. We also observed and reported that the use of the new flask impacted the phenotype of post expansion CD8^+^ T cells, which had a consistently higher expression of a molecule previously associated with clinical response in our clinical trial.^3,7^ This molecule, B-and-T lymphocyte attenuator (BTLA), was recently reported by our group to be expressed by a less-differentiated subtype of CD8^+^ TIL, favoring persistence after transfer in patients treated with TIL ACT.^8^ Given these interesting observations and the major difference in the oxygen availability in the G-Rex vs. the lower oxygen uptake in the traditional T175 flask in a standing position, we decided to investigate the “breathing capacity” or metabolic differences of the cell product expanded in each device.

## Results

### Assessment of TIL rapid expansion in Gas-permeable flask compared to traditional devices

**Figure 1.**
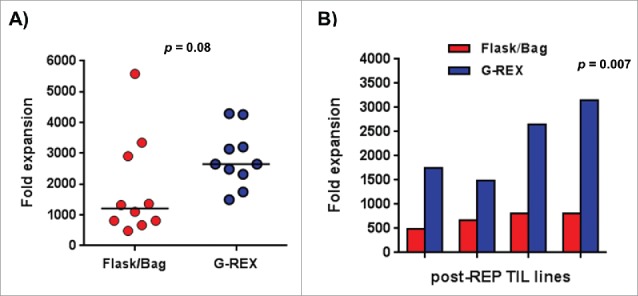
Assessment of TIL growth after rapid expansion in the traditional flask and bag vs. Gas-permeable flask (G-Rex) reveals a trend toward improved TIL expansion when using the G-Rex flask. (**A**) Fold expansion of post-REP TIL lines expanded shows a trend toward a better expansion when using the G-Rex. N = 10. (**B**) The G-Rex flask facilitates the expansion of TIL lines from which the growth is impaired in the REP using the traditional flask and bag devices N = 4. Statistical significance was determined by using a paired *t*-test.

In our endeavor to facilitate the technical aspects of the clinical TIL REP and based on the work that was initiated by Jin et al., we developed culture parameters to use the gas permeable G-Rex flask as a replacement for the typical culture process of seeding of the cells in a T175 flask and moving to 3L gas permeable bags as the cells expand traditionally used in our clinical trial.^3,5^ We assessed the fold expansion on day 14 of the REP and as shown in **Fig. 1A**, the use of a G-Rex throughout the REP showed a trend toward better growth (median = 2,653) compared to the traditional flask/bag process (median = 1,210) (n = 10, *p* = 0.08). The median fold expansion obtained by propagation using the traditional flask/bag process fits within the range of our retrospective analysis of REP fold expansions from our clinical trial using the flask/bag process (**Fig. S1**). This retrospective analysis also served in illustrating what is considered a “good” expansion vs. a “poor” one (**Fig. S1**). Briefly, the REP process typically results in a fold expansion rate from 500 to 2,000, with 1,000 fold expansion being the target value and less than 800 being in the lower range (below the 1 standard of deviation). Interestingly, we observed significantly better growth when using the G-Rex flask for TIL lines that did poorly (lower range) in the traditional devices (**Fig. 1B**, n = 4, *p* = 0.007). This observation explains the “trend” of better growth observed in the G-Rex flask across all samples since good growing TIL lines were not affected by the type of device used (**Fig. 1**). Neither device negatively affected the post-REP viability (>85 %, data not shown). The differential growth of poor growing TIL lines in the two systems used prompted us to explore potential underlying explanations.

### Impact of the use of a Gas-permeable flask on preservation of clonal diversity in propagated TIL

**Figure 2.**
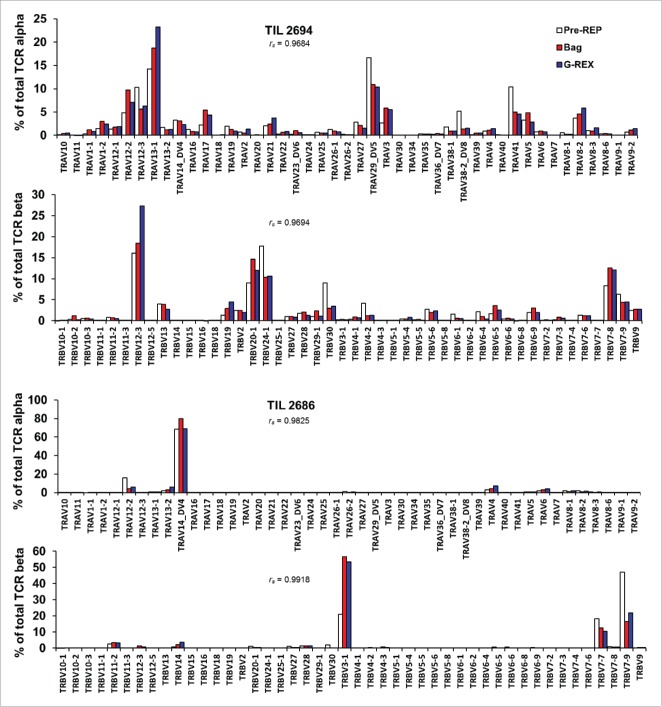
Rapid expansion of TIL in the Gas-permeable flask (G-Rex) does not favor selection and expansion of specific T cell clones. Clonal diversity was evaluated by measurement of the expression of major TCR α and β chain gene by the NanoString nCounter® technology. The analysis of the level of expression of 45 Vα and 46 Vβ genes in RNA isolated from the pre-REP TIL lines in comparison with the different expansion devices demonstrated no difference in the clonal diversity obtained post REP. Two out of 4 TIL lines from 4 melanoma patients are shown. Statistical analysis using a Spearman correlation comparing cells grown in traditional flask and bag (red) vs. Gas-permeable flask (blue) is shown for both Vα and Vβ genes.

We demonstrated above that the G-Rex can rescue the growth of TIL lines that would otherwise expand poorly (**Fig. 1**). Considering that one of the major assets of TIL ACT is clonal diversity allowing simultaneous targeting of numerous antigens, the possibility that a change in manufacturing process could lead to clonal selection was a concern.^9,10^ To investigate this matter, we compared the overall TCR gene diversity of the post-REP products generated with the two different approaches. We also included the pre-REP counterparts to visualize any loss or gain during the REP as it has to our knowledge never been reported. To this end, we evaluated the diversity and abundance of TCR Vβ and Vα chain expression by direct TCR gene expression analysis assay, a non-enzymatic assay based on bar-coded probes.^11^ As shown in **Fig. 2** **and Fig. S2**, slight fluctuations in the frequency of the different TCR Vβ and Vα chains between the pre-expansion and post-expansion TIL were noted but importantly no single Vα or Vβ expression was lost with any of the different devices during the propagation (*r*_*s*_ > 0.9 for all patient samples). In general, the REP did not significantly alter the TCR diversity of the expanded TIL.

### Influence of the Gas-permeable flask on mitochondria respiration and maintenance of a favorable phenotype for TIL ACT

**Figure 3.**
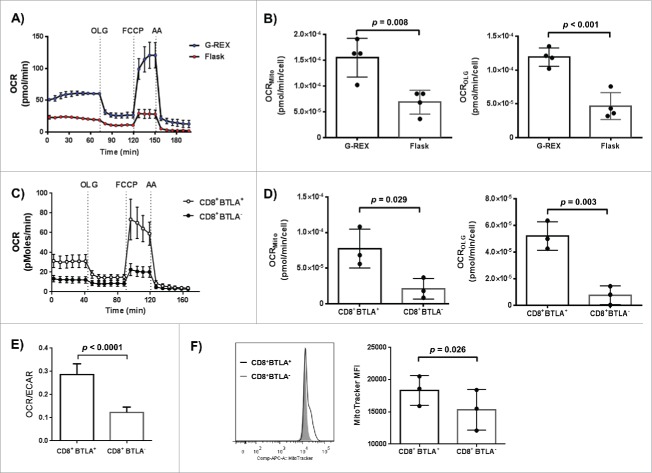
Bioenergetic analysis of melanoma TIL lines to evaluate mitochondrial function. (**A–D**) OCR of 4 TIL lines on day 7 of the REP (**A and B**) or sorted, pre-REP CD8^+^BTLA^+^ and CD8^+^BTLA^−^ TIL line from 3 patients (**C and D**) were determined using a Seahorse XP96 Bioanalyzer. OCR were calculated after 3 min of mix time and 4 min of measurement time. OCR[Mito] is the total mitochondrial OCR, the value just prior to the Oligomycin (OLG) injection minus the non-mitochonddrial OCR component determine by Antimycin (AA) treatment. OCR[OLG] is the component of the OCR that is sensitive to Oligomycin treatment, the rate used by ATP-syntase. The OCR on a per cell basis was determined by dividing the OCR by the seeded TIL count of 250000. (**E**) OCR/ECAR ratio is the OCR[OLG] divided by the ECAR basal which is reflextive of a cell dependence on glycolysis. (**F**) pre-REP TIL were stained using a MitoTracker dye. The MFI of the MitoTracker staining of the CD8^+^BTLA^+^ and CD8^+^BTLA^−^ subset from 3 TIL lines is shown. The histogram shows staining of a representative TIL line. Statistical significance was determined by using a paired *t*-test.

Because of the significant growth advantage for what was considered a poor growing TIL line that was conferred by the G-Rex flask, we hypothesized that a G-Rex may allow cells with higher oxygen consumption rates (OCR) to survive and proliferate. A traditional T175 flask is vented through the cap where the gas-permeable membrane is small in size and located away from the cells. For gas exchange to occur, the gas must first fill the flask, dissolve and equilibrate across the media to finally reach the cells at the bottom. T cells are growing in suspension and settle at the bottom of the flask. We postulated that this setup could create an oxygen gradient that could select for glycolytic cells, those with reduced dependency on mitochondrial respiration. On the other hand, the bottom of the G-Rex may allow for maximal gas exchange at the interface with the cells. Using the Seahorse technology, we evaluated the mitochondrial function of 4 TIL lines from 4 different melanoma patients on day 7 of the REP (G-Rex vs. T175 flask) and on day 14 (G-Rex vs. gas permeable bags). Interestingly, using the same number of live cells for each growing condition, major differences in mitochondria respiration were observed on day 7 of the REP (**Figs. 3A and B**) but not on day 14 (**Fig. S3**). As presented in **Fig. 3A**, basal OCR with drug additions on day 7 of the REP were higher in the TIL propagated in the G-Rex as compared to the traditional flask, showing less dependence on glycolysis.^12^

To evaluate mitochondrial function and the mitochondrial contribution to OCR, small molecules inhibitors (Oligomycin (OLG), FCCP and Antimycin (AA)) were added sequentially. When mitochondrial respiration was forced by addition of FCCP, which disturbs the inhibitory mitochondrial action of the previously added Oligomycin by uncoupling ATP synthesis, TIL expanded in the T175 flask had only a minor increase in oxygen consumption. However, the OCR of the TIL expanded in the G-Rex almost tripled, demonstrating an enhanced mitochondrial capacity for cells expanded in this manner. The addition of AA, a general mitochondrial inhibitor, brought the OCR level to a minimum showing the total contribution of the mitochondria respiration. In addition, the total mitochondrial OCR [basal OCR – minimal level OCR (after AA)] on a “per cell” basis was significantly different between the 2 growth conditions (*p* = 0.008, **Fig. 3B**, left graph). The right graph reinforces the argument by showing a significant difference, again on a “per cell” basis, between the normal ATP generation by mitochondria (basal OCR) and the inhibition of ATP synthesis with Oligomycin (OCR level after OLG) [basal OCR – OCR level after OLG, *p* < 0.001]. The G-Rex grown cells consistently had a higher respiratory capacity than the corresponding T175 flask cells. All together, the metabolic phenotype of melanoma TIL propagated in G-Rexs was consistently more oxidative.

To evaluate the cause of this oxidative phenotype, we considered that the state of cellular differentiation may change the metabolic dependencies of the cells. We previously reported that CD8^+^ TIL rapidly expanded in the G-Rex flask displayed a higher expression of BTLA compared to a traditional flask.^7^ Because of our previous interest in BTLA as a biomarker for clinical response to TIL ACT and our recent study demonstrating enhanced proliferative capacity and persistence *in vivo* of this subset after TIL ACT, we sought to interrogate this population's metabolic phenotype.^3,8^ Thus, since BTLA^+^ cells are believed to be less-differentiated cells and their expansion is promoted when TIL are propagated in G-Rex flasks, we hypothesized that BTLA^+^ TIL could be less glycolytic. To this end, CD8^+^ TIL from three different melanoma patients were sorted into BTLA^+^ and BTLA^−^ subsets to examine their distinct bioenergetic profiles. As shown in **Fig. 3C and D**, CD8^+^BTLA^+^ TIL displayed a higher basal OCR and enhanced mitochondrial function and spare respiratory capacity as compared to their BTLA^−^ counterparts (*p* = 0.029 and *p* = 0.003, respectively). The observation was similar with what was observed in **Fig. 3A and B** when comparing growth devices. To support the observed oxidative shift in metabolism, the OCR to extracellular acidification rate (ECAR) ratio (OCR/ECAR) was evaluated. Consistent with an oxidative phenotype, the OCR/ECAR ratios of BTLA^+^ TIL were higher than the BTLA^−^ counterparts (**Fig. 3E**, *p* < 0.0001). The higher mitochondrial function observed in the BTLA^+^ subset could be attributed to a higher number of mitochondria or an increase mitochondrial membrane potential. To address this question, mitochondria numbers were evaluated using MitoTracker® staining by flow cytometry. As shown in **Fig. 3F**, BTLA^+^ TIL had a significantly higher mitochondrial content (n = 3, *p* = 0.026). Because of the BTLA^+^ TIL enhanced oxidative phosphorylation (OXPHOS) activity and the close proximity to oxygen when grown in a G-Rex, this subset is able to preferentially expand without skewing the clonal repertoire of the TIL.

## Discussion

The impact of metabolism and mitochondrial function on T cell differentiation and persistence is rapidly gaining ground. In this study, we explored the impact of a new cell culture device on rapidly expanding melanoma TIL, demonstrated its ability to rescue poor growth and proposed a rationale behind the preferable expansion of a subset of CD8^+^ T cells that has been shown to correlate with clinical response to TIL ACT.^3,7^ Prior studies demonstrated that this subset, CD8^+^BTLA^+^ T cells, possess a high proliferative capacity, generate their own IL-2 and display enhanced persistence in melanoma patients after TIL ACT.^8^

Importantly, the use of a Gas-permeable device for TIL propagation in the current study did not favor clonal selection, which demonstrates an impact on the metabolism of the TIL product as a whole as opposed to selective expansion of a particular subset. This may appear to contrast with the finding that CD8^+^BTLA^+^ TIL subset is enriched in the end product but since we have shown in the previous work that there is over 90% overlap in the identity of the T cell clones forming CD8^+^BTLA^+^ TIL and CD8^+^BTLA^−^ TIL subsets through the use of high throughput CDR3 sequencing, therefore the data suggests that BTLA marks a state of differentiation to which all clones are subjected.^8^ With the perspective that the vast majority of clones are represented in both BTLA subsets it becomes clear that over representation of the BTLA positive subset will not change the clonal distribution.

As underlined before, TIL are comprised of activated T cells that are enriched in tumor specificity.^10^ As such, these cells have already been activated *in vivo* and switched from dependence on oxidative phosphorylation (naive T cells) to glycolysis (effector T cells).^12,13^ However, it was also demonstrated that the generation of memory T cells is reported to require a higher consumption of oxygen and a higher spare respiratory capacity in the mitochondria.^14,15^ Consistent with our data, one could postulate that favoring oxygen availability could potentially favor expansion of cells that require higher oxygen consumption or at least allow uninhibited expansion of this population. Our Seahorse assessment of the mitochondrial respiration shows higher levels of mitochondrial activity in TIL expanded with the G-Rex, validating this hypothesis. The fact that this observation was only valid for the G-Rex verses T175 flask comparison on day 7, but not when compared to the gas-permeable bag on day 14 of the REP reinforces the importance of oxygen availability which is more readily available in the gas permeable bag. These results also demonstrate the importance of the first 7 d of expansion on the phenotype of the end product. Overall, our data demonstrates that a G-Rex could favor expansion of less-differentiated cell subsets likely due to the high availability of oxygen.

As mentioned previously, the G-Rex flask also favors propagation of BTLA^+^ TIL.^7^ When evaluated separately, BTLA^+^ cells consumed oxygen at higher rates than BTLA^−^ matched cells which would, at least in part, explain their preferential expansion in the G-Rex. Increased OCR, OCR/ECAR ratios, spare respiratory capacity and MitoTracker staining demonstrate that the BTLA^+^ cells have a more oxidative phenotype.^16,17^ High spare respiratory capacity is believed to be a feature of memory T cells that imparts them with better *in vivo* persistence, as opposed to effector T cells.^14,15,18^ We are thus postulating that the high spare respiratory capacity and the high OCR that we observed in TIL grown in high oxygen environment, more specifically the CD8^+^BTLA^+^ cells, could therefore result in enhanced survival and persistence after transfer, which could confer better tumor control. The persistence of CD8^+^BTLA^+^ TIL was previously observed by our group and this study now demonstrates a metabolic mechanism supporting our previous findings.^8^

Overall this study demonstrates a higher proliferative capacity, a positive metabolic shift, and maintenance of polyclonal diversity of TIL cultured in gas-permeable devices. We propose that these devices may have a positive impact on response to TIL ACT through the generation of higher numbers of TIL for infusion that may persist longer *in vivo*.

## Materials and Methods

### Initial propagation of TIL from human melanoma tumors

The TIL that were used in this study were isolated from tumor samples obtained from melanoma patients with stages IIIc and IV disease undergoing surgery at the University of Texas MD Anderson Cancer Center (MDACC) according to Institutional Review Board-approved protocols and patient consent (2004–0069, LAB06–0755 and LAB06–0757). The pre-REP expansion was carried out in 24-well plates from small tumor fragments (3–5 mm^2^).^3^

### Rapid expansion of TIL (REP)

Melanoma TIL initially derived from tumor fragments were propagated by REP with anti-CD3 (OKT3, Orthoclone, 59676–101–01) using pooled allogeneic irradiated PBMC feeder cells (ratio of 1 TIL to 200 feeders), based on the protocol currently used for Phase II clinical trials carried out at MDACC.^3^ The REP was initiated in T175 flasks (Nunc, 178883) for the first 7 d and then transferred into 3L cell culture bags (OriGen, 3000N) for the 7 remaining days. For TIL propagation carried out in the G-Rex, 5×10^6^ TIL were seeded per G-Rex 100M flask (Wilson Wolf Manufacturing, 81300S) on day 0 in a volume of 400 mL and the expansion was completed using the same reagents and feeding schedule than the protocol performed in traditional flask and bags.^3^ The amounts of reagents added were adapted for an optimal use of the G-Rex 100 M flask. Briefly, on day 5 of the REP, 200 mL (+IL-2) of media was added. Counts to assess expansion during the REP were only performed on day 7 where culture from each individual G-Rex flask were either split into 4 flasks or split to seed an average of 100×10^6^cells/flask if growth was not optimal in a maximum of 600 mL of media (+IL-2). Another 600 mL (+IL-2) was added on day 9 and only IL-2 was added on day 11 or 12.

### Direct TCR expression assay

Total RNA was isolated from post-REP TIL expanded in the traditional devices (flaks/bags) or in the G-Rex 100 M using the RNeasy Mini Kit (Qiagen,74106). RNA isolates were quantified using a NanoDrop 1,000 (Thermo Fisher Scientific). Direct TCR expression was measured by the abundance of 45 Vα and 46 Vβ TCR chains and performed using the nCounter™ (model No. NCT-SYST-120) assay system (NanoString Technologies) as described.^11^

### Measurement of mitochondria respiration

The mitochondrial respiration of TIL was measured on day 7 and 14 of the REP using the Seahorse XF96. 2.5 × 10^5^ TIL were taken from either the T175 flask or G-Rex (day 7) or the 3 L bags or G-REX (day 14) and seeded in a well of the Seahorse XF96 PET plate (Seahorse Bioscience) pretreated with CellTak in bicarbonate free DMEM (5 mM glucose, 0.5 mM glutamine, no phenol red, no serum) and incubated at 37°C. In some cases, sorted, pre-REP CD8^+^BTLA^+^ and CD8^+^BTLA^−^ TIL were seeded as above for bioenergetic analysis. The different conditions were tested in triplicates. OCR were calculated by the Seahorse XP96 Bioanalyzer (Seahorse Bioscience) after 3 min of mix time and 4 min of measurement time. Oligomycin (1 ug/mL), FCCP (0.4 uM), and antimycin (1uM) were added sequentially to the wells to assess the mitochondrial function of the TIL.

### Flow cytometry

Bulk pre-REP TIL were stained with anti-CD8^+^-PB (clone RPA-T8, BD Bioscience, 558207), BTLA-PE (clone J168, BD Bioscience), MitoTracker®Deep Red FM (Invitrogen, M22426) and AQUA live/dead dye (Invitrogen, L34957). First, TIL (1×10^6^/mL) were incubated with 100nM of MitoTracker dye in 2 mL of warm FACS buffer incubated in a 24 wp at 37°C for 45 min. Cells were then washed and resuspended in 100 μL FACS buffer containing the mix of antibodies and live/dead dye for a 30 min on ice incubation followed by a final wash. Data acquisition was performed using a FACScanto II cytometer (BD Biosciences) and analysis was done using FlowJo v7.6.5 (Treestar).

### Statistical analysis

For quantitative differences between 2 groups, a paired, 2-tailed Student's *t* test was performed using Graphpad Prism v5.0 (La Jolla, CA). *p* < 0.05 was considered statistically significant. A Spearman's rank correlation test was utilized on housekeeping gene normalized direct TCR expression transcript counts to assess divergence of the TCR repertoire in T cells expanded in either the G-Rex or traditional flask/bag device as previously described.^19^ If the correlation coefficient was greater than 0.8 (*r*_*s*_>0.8), the 2 TCR repertoires were considered to be highly correlated.

## Supplemental Material

Supplemental Material may be downloaded here: publisher's website
