# Supplementary material for: The beneficial effects of a gas-permeable flask for expansion of Tumor-Infiltrating lymphocytes as reflected in their mitochondrial function and respiration capacity
Source: Oncoimmunology. 2015 Jun 5;5(2):e1057386. doi: 10.1080/2162402X.2015.1057386 (PMC4801448; doi:10.1080/2162402X.2015.1057386)
Supplement: 1057386_supplemental_files.zip [file koni-05-02-1057386-s001.zip › 1057386 supplemental files/2015ONCOIMM0119R1-s02.pptx]

## Slide 1
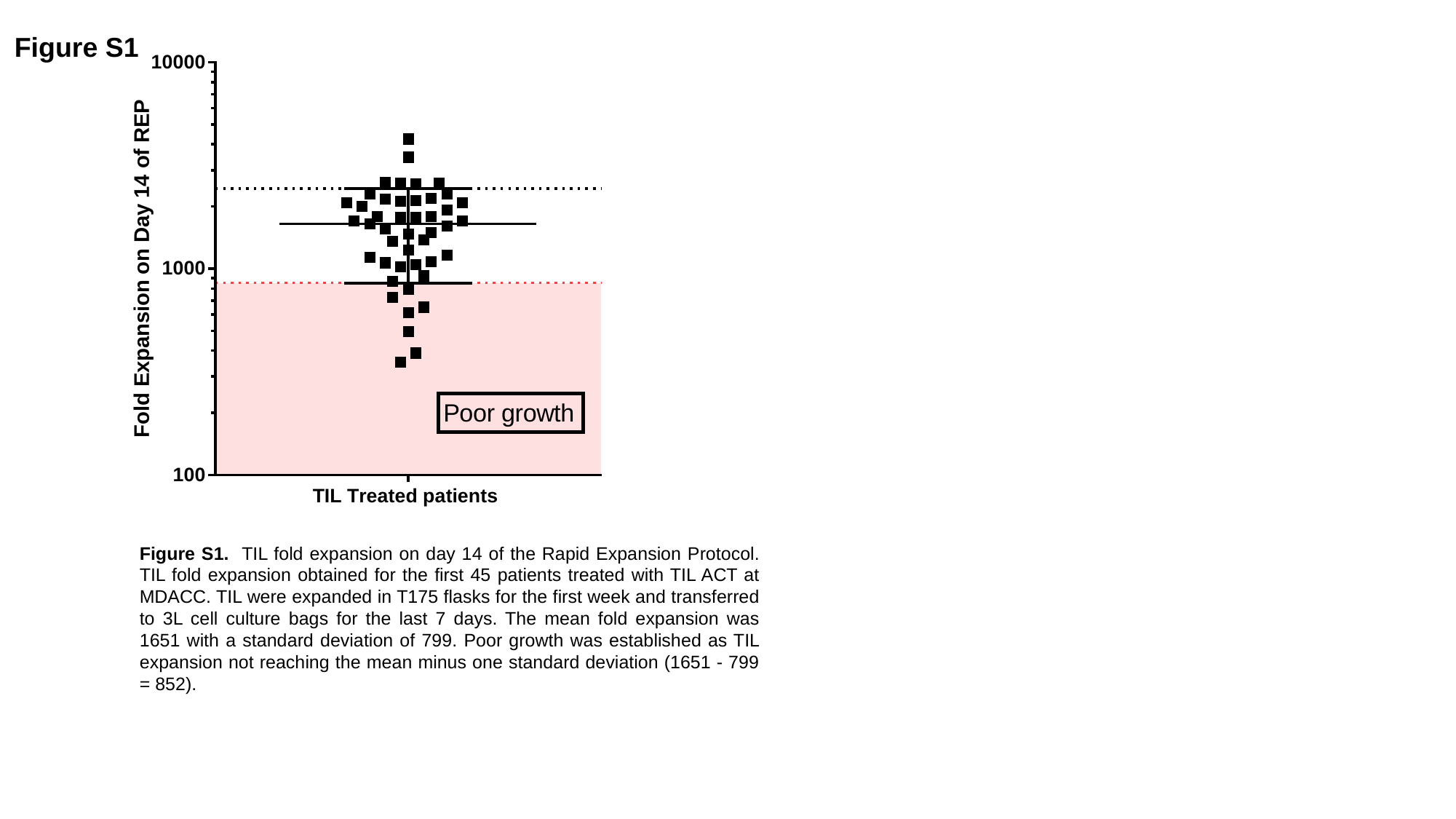

Figure S1
Figure S1. TIL fold expansion on day 14 of the Rapid Expansion Protocol. TIL fold expansion obtained for the first 45 patients treated with TIL ACT at MDACC. TIL were expanded in T175 flasks for the first week and transferred to 3L cell culture bags for the last 7 days. The mean fold expansion was 1651 with a standard deviation of 799. Poor growth was established as TIL expansion not reaching the mean minus one standard deviation (1651 - 799 = 852).
