# Supplementary material for: The beneficial effects of a gas-permeable flask for expansion of Tumor-Infiltrating lymphocytes as reflected in their mitochondrial function and respiration capacity
Source: Oncoimmunology. 2015 Jun 5;5(2):e1057386. doi: 10.1080/2162402X.2015.1057386 (PMC4801448; doi:10.1080/2162402X.2015.1057386)
Supplement: 1057386_supplemental_files.zip [file koni-05-02-1057386-s001.zip › 1057386 supplemental files/2015ONCOIMM0119R1-s03.pptx]

## Slide 1
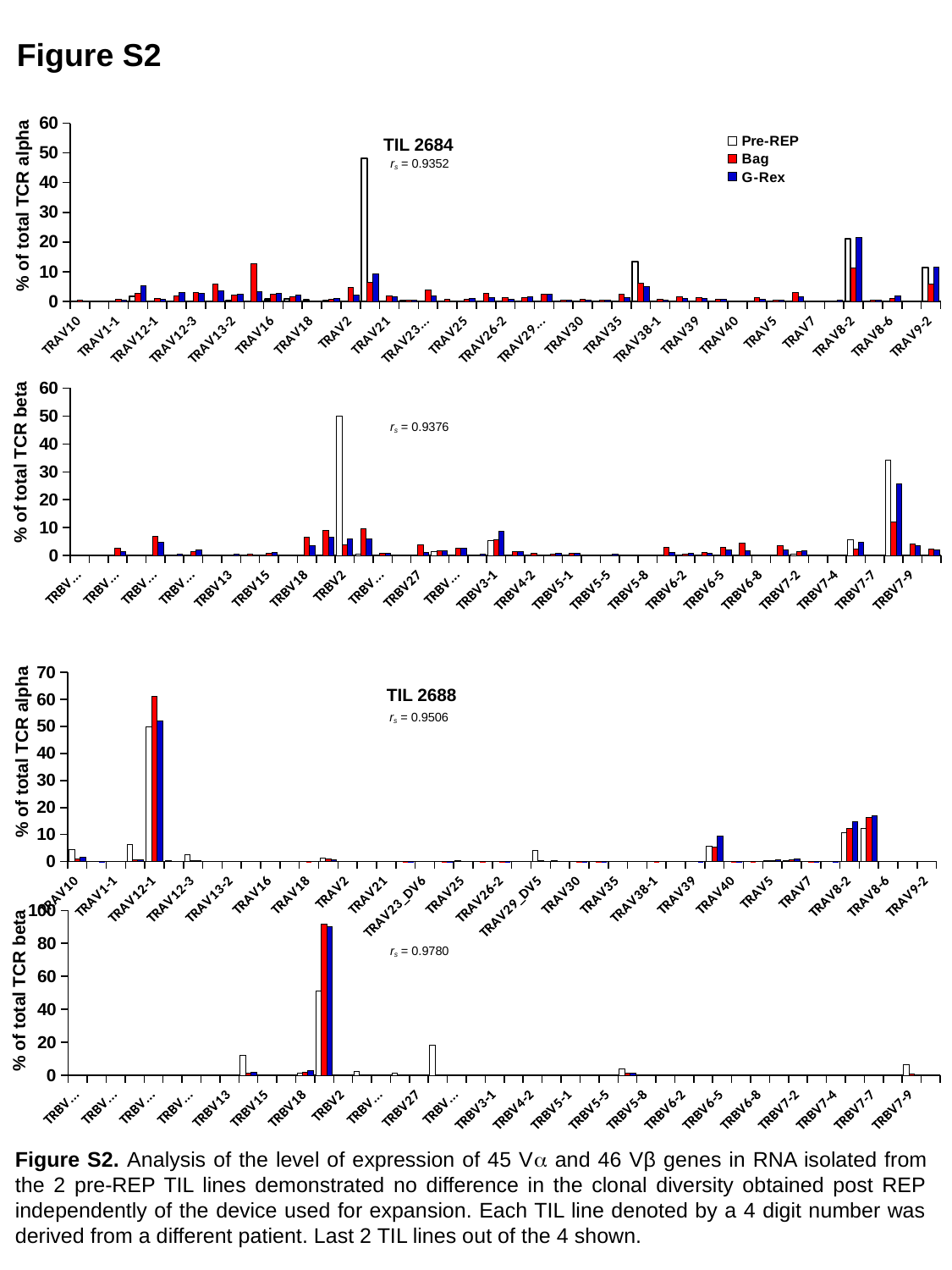

Figure S2
### Chart
| Category | Pre-REP | Bag | G-Rex |
|---|---|---|---|
| TRAV10 | 0.0037306591122142564 | 0.5054134060016209 | 0.1951808288732075 |
| TRAV11 | -0.0032544047574635006 | -0.0031084343536956806 | 0.0002163067922495835 |
| TRAV1-1 | 0.0030956533058799146 | 0.6960640463616227 | 0.35784353664489427 |
| TRAV1-2 | 1.6328380552629675 | 2.8299817052870866 | 5.255029313175465 |
| TRAV12-1 | 0.02405084491491319 | 0.8924810576965961 | 0.6534628193859917 |
| TRAV12-2 | 0.11803170425239572 | 1.8987127928291572 | 3.12830093177756 |
| TRAV12-3 | 0.030083400075089434 | 2.8930513878258393 | 2.5956094047309186 |
| TRAV13-1 | 0.07421630361532616 | 5.916792167826623 | 3.6105208739659647 |
| TRAV13-2 | 0.3313936551807345 | 2.1272052427124106 | 2.312103302355798 |
| TRAV14_DV4 | 0.08310638490400694 | 12.61515285162812 | 3.346914996477806 |
| TRAV16 | 0.7149371622066768 | 2.371915610962772 | 2.774423019657241 |
| TRAV17 | 0.8527334221812288 | 1.690042243172369 | 2.2535562639202444 |
| TRAV18 | 0.6171462680311882 | 0.20376012437341395 | 0.25372786730876146 |
| TRAV19 | 0.3977517619426732 | 0.7241751048646096 | 0.9467748296764269 |
| TRAV2 | 0.02405084491491319 | 4.617917105714248 | 2.03609583544533 |
| TRAV20 | 48.16130099989601 | 6.304580615893469 | 9.405812451916805 |
| TRAV21 | 0.038020972654268695 | 1.783385373329723 | 1.6303042931851106 |
| TRAV22 | 0.17010218037181174 | 0.47297756926740503 | 0.4841667033186511 |
| TRAV23_DV6 | 0.03230592039725962 | 3.7634130068606297 | 1.9960069766150734 |
| TRAV24 | 0.0030956533058799146 | 0.7767932400112263 | 0.25228582202709754 |
| TRAV25 | 0.01770078685156977 | 0.7858031946596197 | 0.9372573308174453 |
| TRAV26-1 | 0.048498568458785335 | 2.7269078241094675 | 1.3320893289370184 |
| TRAV26-2 | 0.039290984266937376 | 1.1656628826358801 | 0.763635078905113 |
| TRAV27 | 0.0097632142723905 | 1.2788279130196998 | 1.4699488578640862 |
| TRAV29_DV5 | 0.0227808333022445 | 2.5856317352226608 | 2.5087982787747527 |
| TRAV3 | 0.0097632142723905 | 0.5522651701732659 | 0.5626139666411667 |
| TRAV30 | 0.1075541084478791 | 0.6236240109885407 | 0.4316762550660855 |
| TRAV34 | 0.004365664918548598 | 0.3569293533960996 | 0.4086035305594632 |
| TRAV35 | 0.08501140232300997 | 2.3484897288769493 | 1.3701593243729449 |
| TRAV36_DV7 | 13.409338236636497 | 6.2598912408374385 | 4.90057458294248 |
| TRAV38-1 | 0.00563567653121728 | 0.6319131692650624 | 0.4980103380226245 |
| TRAV38-2_DV8 | 0.01706578104523543 | 1.5538117288886626 | 1.0361816371395882 |
| TRAV39 | 0.029448394268755087 | 1.2204434068981111 | 1.0105132311259708 |
| TRAV4 | 0.051673597490457045 | 0.8402233207359151 | 0.7183548570608668 |
| TRAV40 | -0.0026193989511291587 | 0.06608801734596467 | 0.12625106440967354 |
| TRAV41 | 0.014843260723065233 | 1.25936641097917 | 0.6704789537096256 |
| TRAV5 | 0.006270682337551623 | 0.5648791066810165 | 0.4155253479114499 |
| TRAV6 | 0.018970798464238457 | 3.001170843606558 | 1.6346304290301026 |
| TRAV7 | -0.002301896047961988 | 0.0004955475056616301 | 0.0028119882992445853 |
| TRAV8-1 | 0.0304009029782566 | 0.24808910124350889 | 0.33390558496927375 |
| TRAV8-2 | 21.085288423606023 | 11.297537083847088 | 21.482653276795556 |
| TRAV8-3 | 0.08310638490400694 | 0.5104589806047211 | 0.5352151062895527 |
| TRAV8-6 | 0.13295434070125275 | 1.1000704127955772 | 1.805368590379107 |
| TRAV9-1 | -0.0016668902416276468 | 0.06284443367254311 | 0.015790395834219595 |
| TRAV9-2 | 11.468125486672417 | 5.877869163745563 | 11.540616286892027 |TIL 2684
rs = 0.9352
% of total TCR alpha
### Chart
| Category | Pre-REP | Bag | G-Rex |
|---|---|---|---|
| TRBV10-1 | -0.006919358456171832 | 0.02196796721681895 | 0.017602479103643816 |
| TRBV10-2 | 0.05416187481210365 | 0.2272812862209935 | 0.20311363612408162 |
| TRBV10-3 | 0.053207480542286835 | 2.543751124550702 | 1.5050646653947906 |
| TRBV11-1 | 0.015986104019431468 | 0.07162254708467637 | 0.14493059142221706 |
| TRBV11-2 | 0.12765023358799757 | 6.7973074943545795 | 4.899075606336892 |
| TRBV11-3 | 0.03698277795540116 | 0.2507137845856004 | 0.591000600803179 |
| TRBV12-3 | 0.2870340766474039 | 1.4028116208454389 | 2.037987625562594 |
| TRBV12-5 | 0.0016701899721794076 | 0.11904546044161889 | 0.34224700388941 |
| TRBV13 | 0.034119595145950754 | 0.2373237855201107 | 0.38061408409136416 |
| TRBV14 | 0.01121413267034745 | 0.42534168906469444 | 0.29755567969812274 |
| TRBV15 | 0.017894892559065078 | 0.7054158361845195 | 1.2019225701727572 |
| TRBV16 | 0.01503170974961467 | 0.1179296271861614 | 0.11963361546488463 |
| TRBV18 | 0.091383251334959 | 6.654480837656022 | 3.5916435656087606 |
| TRBV19 | 0.10474477111239427 | 8.90176901414737 | 6.507120044691324 |
| TRBV2 | 49.865907605090754 | 3.9681122751421634 | 5.972932235725654 |
| TRBV20-1 | 0.58385069456043 | 9.607533548224218 | 6.018045176182897 |
| TRBV24-1 | 0.05225308627247003 | 0.7863137472051862 | 0.8595701622168583 |
| TRBV25-1 | 0.005487767051446625 | 0.09170754568291085 | 0.2216647518261254 |
| TRBV27 | 0.07515854874807333 | 3.7494089570724984 | 1.2196304533428899 |
| TRBV28 | 1.523929050329982 | 1.7264032649281051 | 1.6661220789898075 |
| TRBV29-1 | 0.09329203987459261 | 2.7624544426203665 | 2.6463798973364385 |
| TRBV30 | 0.06370581751027167 | 0.33607502862809685 | 0.5880492869414902 |
| TRBV3-1 | 5.327190215549947 | 5.756235067012759 | 8.679287046894268 |
| TRBV4-1 | 0.07993052009715736 | 1.4864991150047488 | 1.400925447703772 |
| TRBV4-2 | 0.014077315479797863 | 0.7712499982565103 | 0.2153405078367923 |
| TRBV4-3 | 0.061797028970638074 | 0.5597995963473197 | 0.6799616329197979 |
| TRBV5-1 | 0.03507398941576756 | 0.7997037462706759 | 0.6660482961432652 |
| TRBV5-4 | 0.012168526940164254 | 0.2953471148038992 | 0.2338916235388361 |
| TRBV5-5 | 0.06656900031972209 | 0.22672336959326475 | 0.3721817587722534 |
| TRBV5-6 | 0.009305344130713843 | 0.2953471148038992 | 0.17191403244337164 |
| TRBV5-8 | 0.020758075368515493 | 0.025873383610920096 | 0.0319374321461322 |
| TRBV6-1 | 0.020758075368515493 | 3.016306508236941 | 1.0113520179608528 |
| TRBV6-2 | 0.019803681098698688 | 0.5899270942446714 | 0.8220463145468151 |
| TRBV6-4 | 0.026484440987416317 | 1.0764303936241284 | 0.7107396203345525 |
| TRBV6-5 | 0.057025057621554055 | 3.0347177569519888 | 1.9190918385631317 |
| TRBV6-6 | 0.042709143574301986 | 4.409424327675593 | 1.7601425062978928 |
| TRBV6-8 | 0.002624584241996212 | 0.08724421266108097 | 0.013807932710043953 |
| TRBV6-9 | 0.17155236999957058 | 3.521778972959175 | 2.1062894606473916 |
| TRBV7-2 | 0.4826849019598487 | 1.5043524470920686 | 1.8301308064465127 |
| TRBV7-3 | 0.005487767051446625 | 0.07441213022332005 | 0.03109419961422111 |
| TRBV7-4 | 0.0016701899721794076 | 0.06492754755193154 | 0.05133178038008706 |
| TRBV7-6 | 5.687951249540699 | 2.2056536481470888 | 4.830773771252094 |
| TRBV7-7 | 0.08088491436697416 | 0.23286045249828086 | 0.1369198823690618 |
| TRBV7-8 | 34.28828433314087 | 12.070735459646585 | 25.67506034382806 |
| TRBV7-9 | 0.19922980382425792 | 4.088622266731569 | 3.52924435824734 |
| TRBV9 | 0.1782331298882882 | 2.3010573914887025 | 2.088581577477259 |rs = 0.9376
% of total TCR beta
### Chart
| Category | Pre-REP | Bag | G-Rex |
|---|---|---|---|
| TRAV10 | 4.298373675348143 | 0.9215261440035711 | 1.679783253773707 |
| TRAV11 | -0.0026594516210757344 | -0.0009298952008108686 | 0.005568042154153285 |
| TRAV1-1 | 0.04996969624863353 | 0.024363254261244763 | 0.021321527273221116 |
| TRAV1-2 | 6.188543922243235 | 0.4766642799356514 | 0.6194107381730032 |
| TRAV12-1 | 49.9502402604583 | 61.058220738522785 | 51.917104074856226 |
| TRAV12-2 | 0.3612655070524458 | 0.15529249853541507 | 0.15332486809851364 |
| TRAV12-3 | 2.4238801959036045 | 0.28175824584569326 | 0.4559004271095751 |
| TRAV13-1 | 0.07236507832085026 | 0.10321836729000643 | 0.13050947585710504 |
| TRAV13-2 | 0.0488499271450227 | 0.03924157747421866 | 0.05011237938737957 |
| TRAV14_DV4 | 0.043251081626968516 | 0.11214536121779077 | 0.12942302860751417 |
| TRAV16 | 0.10371861322195364 | 0.07792521782795081 | 0.07890323150153801 |
| TRAV17 | 0.14850937736638706 | 0.10916969657519598 | 0.15006552634974096 |
| TRAV18 | 0.08356276935695861 | 0.013948428012163032 | 0.044136919514629704 |
| TRAV19 | 1.3802553913383062 | 0.8337440370470248 | 0.7660811168677728 |
| TRAV2 | 0.09364069128945612 | 0.06602255925757168 | 0.09085415124703775 |
| TRAV20 | 0.020855699554751812 | 0.07643738550665341 | 0.04739626126340236 |
| TRAV21 | 0.019735930451140977 | 0.08536437943443775 | 0.03979113051626616 |
| TRAV22 | 0.024215006865584324 | 0.005021434084378691 | 0.00665448940374417 |
| TRAV23_DV6 | 0.030933621487249333 | 0.021387589618649984 | 0.04142080139065248 |
| TRAV24 | 0.009658008518643457 | 0.010972763369568253 | 0.01317317290128948 |
| TRAV25 | 0.42509234595826345 | 0.06007122997238213 | 0.05337172113615222 |
| TRAV26-1 | 0.025334775969195155 | 0.005021434084378691 | 0.021864750898016556 |
| TRAV26-2 | 0.019735930451140977 | 0.007997098726973472 | 0.018062185524448462 |
| TRAV27 | 0.008538239415032622 | 0.02287542193994737 | 0.021321527273221116 |
| TRAV29_DV5 | 4.0486651652429275 | 0.170170821748389 | 0.11421276711324176 |
| TRAV3 | 0.4295714223727068 | 0.10916969657519598 | 0.08759480949826509 |
| TRAV30 | 0.0006998556897567721 | 0.005021434084378691 | 0.01317317290128948 |
| TRAV34 | 0.004059163000589279 | 0.007997098726973472 | 0.0023087004053806304 |
| TRAV35 | 0.036532467005303514 | 0.03031458354643432 | 0.03055632889474364 |
| TRAV36_DV7 | 0.09364069128945612 | 0.04221724211681344 | 0.03707501239228895 |
| TRAV38-1 | 0.024215006865584324 | 0.012460595690865642 | 0.02675376352117554 |
| TRAV38-2_DV8 | 0.0611673872847419 | 0.0630468946149769 | 0.06695231175603827 |
| TRAV39 | 0.04213131252335769 | 0.02733891890383954 | 0.015346067400471248 |
| TRAV4 | 5.56707206973922 | 5.216898055589136 | 9.44733786472374 |
| TRAV40 | 0.004059163000589279 | 0.003533601763081301 | 0.0023087004053806304 |
| TRAV41 | 0.009658008518643457 | 0.010972763369568253 | 0.020235080023630232 |
| TRAV5 | 0.3657445834668892 | 0.33383237709110186 | 0.6373371177912529 |
| TRAV6 | 0.4452481898232585 | 0.7325714391988026 | 1.0061859590273583 |
| TRAV7 | 0.009658008518643457 | 0.010972763369568253 | 0.012086725651698596 |
| TRAV8-1 | 0.015256854036697638 | 0.028826751225136935 | 0.016432514650062135 |
| TRAV8-2 | 10.676578489515464 | 12.304931234249901 | 14.898586939546002 |
| TRAV8-3 | 12.22521915980925 | 16.186685760514795 | 16.86722935580469 |
| TRAV8-6 | 0.036532467005303514 | 0.03031458354643432 | 0.029469881645152757 |
| TRAV9-1 | 0.05892784907752023 | 0.08387654711314037 | 0.08053290237592435 |
| TRAV9-2 | 0.017496392243919308 | 0.021387589618649984 | 0.03272922339392541 |TIL 2688
rs = 0.9506
% of total TCR alpha
### Chart
| Category | Pre-REP | Bag | G-Rex |
|---|---|---|---|
| TRBV10-1 | 0.021538212200437588 | 0.015171617770426648 | 0.020532333716352404 |
| TRBV10-2 | 0.013008227170561314 | 0.03183064904775787 | 0.020532333716352404 |
| TRBV10-3 | 0.08807209543347251 | 0.05324940354718373 | 0.07402942339932564 |
| TRBV11-1 | 0.01812621818848708 | 0.0008924481041427441 | 0.01224405221617345 |
| TRBV11-2 | 0.14266399962468063 | 0.39118975231590275 | 0.3219243882683143 |
| TRBV11-3 | 0.04201017627214064 | 0.005652171326237379 | 0.004709250852374405 |
| TRBV12-3 | 0.5691632511184943 | 0.05324940354718373 | 0.06272722135362709 |
| TRBV12-5 | 0.0027722451347097885 | -0.0014874135069045734 | 0.0032022905796145955 |
| TRBV13 | 0.04201017627214064 | 0.04848968032508909 | 0.02128581385273231 |
| TRBV14 | 11.97034124205112 | 1.5668413881732777 | 2.008966413622921 |
| TRBV15 | 0.024950206212388092 | 0.06514871160242032 | 0.08834554599054383 |
| TRBV16 | 0.007890236152635552 | 0.0008924481041427441 | 0.004709250852374405 |
| TRBV18 | 1.2345020834488436 | 1.9476192459408483 | 2.8656733286868725 |
| TRBV19 | 51.19291840642819 | 91.46611374548569 | 90.13449620434382 |
| TRBV2 | 0.040304179266165384 | 0.02469106421461592 | 0.023546254261872023 |
| TRBV20-1 | 2.4167580085896945 | 0.4340272613147545 | 0.5261175052272685 |
| TRBV24-1 | 0.4855693978257068 | 0.06752857321346763 | 0.09211294667244335 |
| TRBV25-1 | 1.2737400145862743 | 0.0008924481041427441 | 0.004709250852374405 |
| TRBV27 | 0.09830807746932405 | 0.20318068504316472 | 0.18479100344717164 |
| TRBV28 | 18.29447214320139 | 0.5173224177014106 | 0.23452069244824536 |
| TRBV29-1 | 0.12219203555297758 | 0.08180774287975154 | 0.09437338708158308 |
| TRBV30 | 0.028362200224338607 | 0.015171617770426648 | 0.02957409535291126 |
| TRBV3-1 | 0.023244209206412843 | 0.062768849991373 | 0.03710889671671031 |
| TRBV4-1 | 0.25184780800709694 | 0.1341646983227925 | 0.17122836099233338 |
| TRBV4-2 | 0.05224615830799217 | 0.05086954193613641 | 0.09135946653606344 |
| TRBV4-3 | -0.0006397488772407204 | -0.0038672751179518908 | -0.0020720703750447384 |
| TRBV5-1 | 0.03177419423628911 | 0.03897023388089983 | 0.028820615216531356 |
| TRBV5-4 | 0.006184239146660297 | 0.012791756159379332 | 0.020532333716352404 |
| TRBV5-5 | 0.17337194573223522 | 0.06514871160242032 | 0.06950854258104622 |
| TRBV5-6 | 3.8549134846268345 | 1.1527454678510443 | 1.2848720025618325 |
| TRBV5-8 | 0.0027722451347097885 | 0.0008924481041427441 | 0.0009418501704748809 |
| TRBV6-1 | 0.035186188248239623 | 0.046109818714041774 | 0.0356019364439505 |
| TRBV6-2 | 0.011302230164586062 | 0.03183064904775787 | 0.017518413170832785 |
| TRBV6-4 | 0.0027722451347097885 | 0.010411894548332013 | 0.01224405221617345 |
| TRBV6-5 | 0.059070146331893184 | 0.13654455993383985 | 0.1478704767645563 |
| TRBV6-6 | 0.009596233158610806 | 0.058009126769278364 | 0.07628986380846535 |
| TRBV6-8 | 0.007890236152635552 | 0.008032032937284696 | 0.007723171397894024 |
| TRBV6-9 | 0.06930612836774472 | 0.13654455993383985 | 0.18102360276527213 |
| TRBV7-2 | 0.01812621818848708 | 0.02469106421461592 | 0.031834535762050975 |
| TRBV7-3 | 0.024950206212388092 | 0.03897023388089983 | 0.04313673780774955 |
| TRBV7-4 | 0.038598182260190135 | 0.003272309715190062 | 0.009983611807033737 |
| TRBV7-6 | 0.011302230164586062 | 0.01755147938147397 | 0.01450449262531317 |
| TRBV7-7 | 0.007890236152635552 | -0.0014874135069045734 | 0.005462730988754309 |
| TRBV7-8 | 0.08636609842749725 | 0.14130428315593446 | 0.1004012281726223 |
| TRBV7-9 | 6.471912891792875 | 0.62441619019854 | 0.5487219093186655 |
| TRBV9 | 0.6203431612977519 | 0.21983971632049595 | 0.23226025203910566 |rs = 0.9780
% of total TCR beta
Figure S2. Analysis of the level of expression of 45 Vα and 46 Vβ genes in RNA isolated from the 2 pre-REP TIL lines demonstrated no difference in the clonal diversity obtained post REP independently of the device used for expansion. Each TIL line denoted by a 4 digit number was derived from a different patient. Last 2 TIL lines out of the 4 shown.
