# Supplementary material for: The beneficial effects of a gas-permeable flask for expansion of Tumor-Infiltrating lymphocytes as reflected in their mitochondrial function and respiration capacity
Source: Oncoimmunology. 2015 Jun 5;5(2):e1057386. doi: 10.1080/2162402X.2015.1057386 (PMC4801448; doi:10.1080/2162402X.2015.1057386)
Supplement: 1057386_supplemental_files.zip [file koni-05-02-1057386-s001.zip › 1057386 supplemental files/2015ONCOIMM0119R1-s04.pptx]

## Slide 1
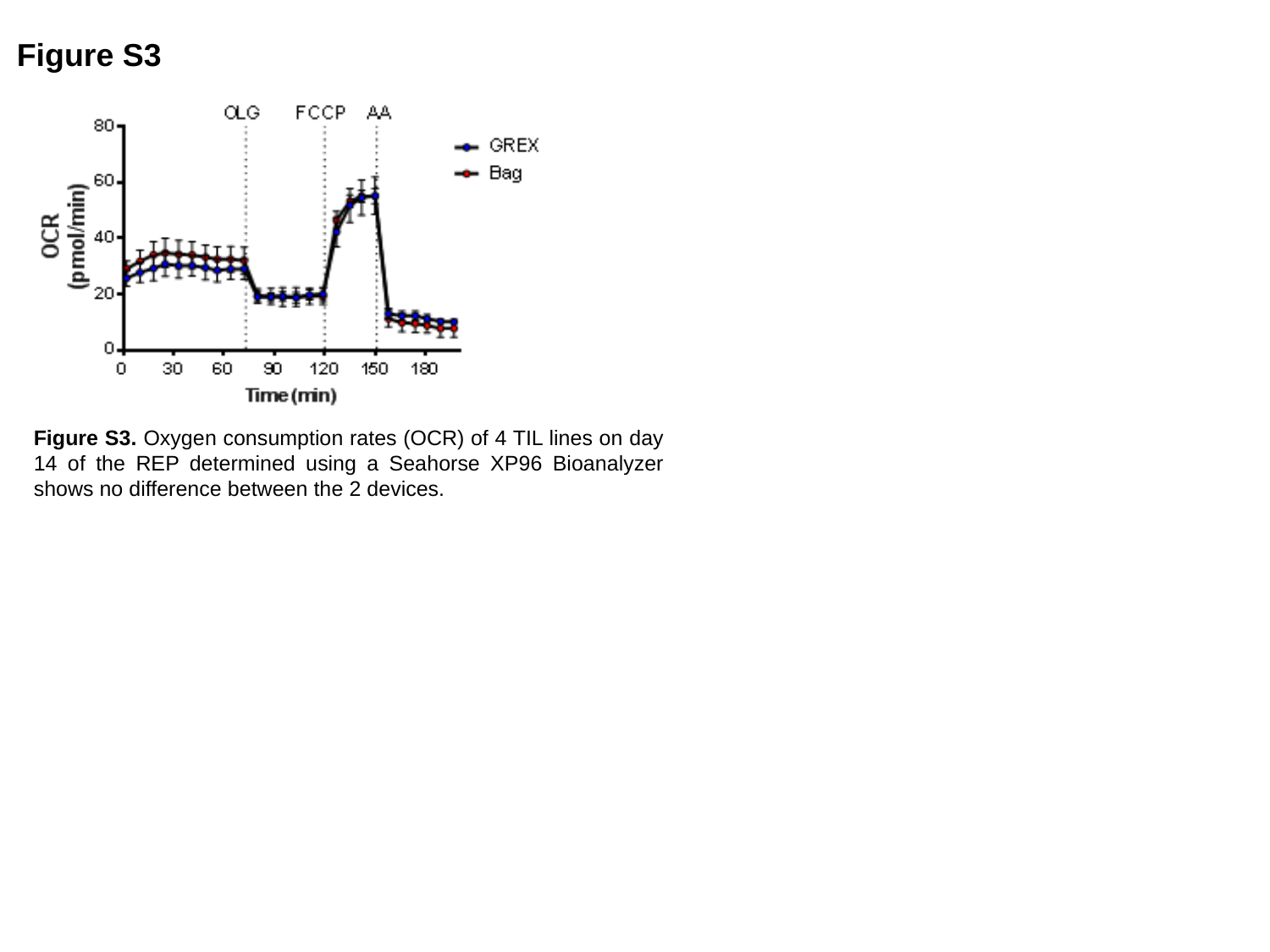

Figure S3
Figure S3. Oxygen consumption rates (OCR) of 4 TIL lines on day 14 of the REP determined using a Seahorse XP96 Bioanalyzer shows no difference between the 2 devices.
